# Practical Notes on Diagnosis and Treatment

**Published:** 1906-07-28

**Authors:** 


### PRACTICAL NOTES ON DIAGNOSIS AND TREATMENT.

**Cold Compresses in Hay Fever.**

Cold compresses, wrung out of ice-cold water and continuously applied to the forehead and face, have given relief in hay fever when many other remedies have failed.

**Ointments for the Eye.**

These should always be made with yellow, not with white, vaseline. The latter, owing to the methods employed in preparation, has irritating qualities.—*Mr. Marcus Gunn.*

**Cardiac Dilatation in Early Life.**

In boys who suffer from cardiac dilatation after strain it is not uncommon to find that after a few minutes' active exercise—for example, hopping on one foot—the apex beat retreats within the nipple line and the systolic murmur diminishes in intensity. The exercise acts as a stimulus, and the heart consequently contracts more completely and successfully. But prolonged exertion exhausts the cardiac muscle, and the evidences of dilatation therefore become more conspicuous. In these cases Oertel's treatment by systematised hill climbing proves beneficial.—*Sir Wm. Broadbent.*

**Calcium Chloride in Chilblains.**

In a series of twenty cases of chilblains, some in the stage of ulceration, occurring in children in the Swansea Deaf and Dumb Institution, Dr. G. Arbour Stephens found that prompt improvement followed the administration of calcium chloride in ten-grain doses.

**Bladder Symptoms and Nervous Disease.**

In cases where there is any difficulty of micturition or other suggestion of bladder disturbance never forget to examine the nerve reflexes, for in a good many cases of spinal cord disease bladder troubles are marked before the nervous condition is very apparent.—*Mr. John Pardoe.*

**Sciatica and Lumbago.**

In treating these conditions Dr. Brindley James advocates a preliminary dry cupping, followed by the subcutaneous injection at the site of the pain of 10 min., increased gradually to 30, if necessary, of sulphuric ether. After a brisk purgative he orders, every four hours, sodium salicylate 5 grs.; spirit of chloroform and tinct. of ginger, of each 10 min.; compound infusion of gentian, 1 oz.

